# Separate and Synergic Effects of *Lactobacillus uvarum* LUHSS245 and Arabinogalactan on the *In Vitro* Antimicrobial Properties as Well as on the Fecal and Metabolic Profile of Newborn Calves

**DOI:** 10.3390/ani10040593

**Published:** 2020-03-31

**Authors:** Paulina Zavistanaviciute, Vita Lele, Ramūnas Antanaitis, Mindaugas Televičius, Modestas Ruzauskas, Qendrim Zebeli, Elena Bartkiene

**Affiliations:** 1 Institute of Animal Rearing Technologies, Faculty of Animal Sciences, Lithuanian University of Health Sciences, Mickeviciaus str. 9, LT-44307 Kaunas, Lithuania; paulina.zavistanaviciute@lsmuni.lt (P.Z.); vita.lele@lsmuni.lt (V.L.); 2 Department of Food Safety and Quality, Faculty of Veterinary Medicine, Lithuanian University of Health Sciences, Mickeviciaus str. 9, LT-44307 Kaunas, Lithuania; 3 Large Animal Clinic, Faculty of Veterinary Medicine, Lithuanian University of Health Sciences, Mickeviciaus str. 9, LT-44307 Kaunas, Lithuania; ramunas.antanaitis@lsmuni.lt (R.A.); mindaugas.televicius@lsmuni.lt (M.T.); 4 Institute of Microbiology and Virology, Faculty of Veterinary Medicine, Lithuanian University of Health Sciences, Mickeviciaus str. 9, LT-44307 Kaunas, Lithuania; modestas.ruzauskas@lsmuni.lt; 5 Department of Anatomy and Physiology, Faculty of Veterinary Medicine, Lithuanian University of Health Sciences, Mickeviciaus str. 9, LT-44307 Kaunas, Lithuania; 6 Institute of Animal Nutrition and Functional Plant Compounds, University of Veterinary Medicine Vienna, Veterinarpl. 1, 1210 Vienna, Austria; qendrim.zebeli@vetmeduni.ac.at

**Keywords:** newborn calves, lactic acid bacteria, arabinogalactan, blood and feces parameters, antimicrobial properties, resistance to antibiotics

## Abstract

**Simple Summary:**

Diarrhea is common problem for young calves. It causes economic losses to cattle producers because for a newborn calf, diarrhea can be fatal. For this reason, calf diarrhea is an expensive disorder, often requiring prolonged medical treatment. Furthermore, treatment often requires use of drugs and antibiotics, increasing public concerns of excessive usage of drugs in dairy farming, and the development of antibiotic resistance. Therefore, prevention remains the best option, and the preventative strategies against newborn diarrhea aim to increase the immunity and the gut health status early after birth. One common prophylactic strategy against diarrhea is the use health-enhancing supplements in the feed. Our hypothesis is that a combination of different origins and mechanisms of action (lactic acid bacteria as an antimicrobial agent and arabibogalactan as a prebiotic for good microbiota stabilization), can lead to improvement in newborn calves’ health parameters. In this study, the lactic acid bacteria strain, LUHS245, effectively inhibited the growth of pathogenic bacteria, as well being non-resistant to all the tested antibiotics. LUHS245, arabinogalactan, and its combination used for newborn calf feeding showed a desirable positive effect on newborn calf health parameters and it can be recommended in dairy farms for diarrhea prophylaxis.

**Abstract:**

In this study, arabinogalactan (ARB) and *Lactobacillus uvarum* LUHS245 antimicrobial properties against pathogenic bacteria (*Klebsiella pneumoniae, Pseudomonas aeruginosa* 17-331, *Acinetobacter baumanni* 17-380, *Proteus mirabilis*, MRSA M87fox, *Enterococcus faecalis* 86, *Enterococcus faecium* 103, *Bacillus cereus* 18 01, and *Streptococcus mutans*) and resistance to antibiotics were evaluated and the role of their supplementation on the main metabolic and fecal variables of newborn calves was established. The animal trial involved 48 Holstein female calves randomly allocated in four homogeneous groups of 12 animals each, on the basis of body weight in the second day of life. Calves were fed with a standard milk replacer diet from the second day of life until 14th day, either unsupplemented or supplemented with 50 mL of LUHS245 (≥7.5 log_10_ CFU mL^−1^), 30 g of ARB, or with both (50 mL of LUHS245 and 30 g ARB). In vitro data showed that the LUHS245 inhibited the growth of *Salmonella enterica* and *Bacillus cereus* (inhibition zones 13.0 and 21.3 mm, respectively). Supplementation of LUHS245 and ARB either alone or together, lowered total bacterial count in the feces and reduced lactate and serum alanine aminotransferase concentrations in blood. This study showed that LUHS245 supplementation alone or together with ARB seemed to have some positive influence on certain health parameters in newborn calves. Further research with larger cohorts of animals is warranted to validate the beneficial effects of the tested supplements.

## 1. Introduction

Diarrhea is common in young calves, remaining the major cause of productivity and economic losses to cattle producers worldwide [1]. In the neonatal calf, diarrhea can be fatal. Calves often suffer from dehydration and the resulting acidosis may cause anorexia and ataxia, increasing the odds of other health complications [2]. Calf diarrhea is an expensive disorder, difficult to treat, often requiring prolonged medical treatment, and affecting the welfare of the young calves. Furthermore, treatment often requires use of drugs and antibiotics, increasing public concerns of excessive usage of drugs in dairy farming, and the development of antibiotic resistance [3]. Resistance to antibiotics may be intrinsic; e.g., inherent to bacterial genus or species, or acquired, either through mutations or through transfer of antibiotic resistance genes from other bacteria [4]. Also, most of the calves recovering from diarrhea still have depressed growth rates, are susceptible to other diseases, and often do not reach the breeding age on time. Therefore, prevention remains the best option, and the preventative strategies against newborn diarrhea aim to increase the immunity and the gut health status early after birth. 

One common prophylactic strategy of diarrhea is the use of health-enhancing supplements in the feed (commonly milk replacers) of calves soon after the birth, aimed at stimulating gut mucosal immunity and establishing the gut microbiome at this early phase. In this respect, research during the last decades has recommended several such supplements including probiotics, prebiotics, and essential oils or herbal extracts, which have also been successful in the replacement of the antibiotics [5]. Accordingly, lactic acid bacteria (LAB) offer various advantages as potential probiotics and can be considered as alternatives to antibiotics in food animal production [6]. Common pathogenic bacteria such *Escherichia coli*, *Salmonella enterica*, *Clostridium perfringens*, *Aeromonas salmonicida*, and *Pseudomonas* spp. can cause infection soon after birth, and LAB can be used to control them, as well as to improve animal growth [6]. The LAB can limit the distribution of pathogenic bacteria by mechanisms involving production of inhibitory compounds such as bacteriocins, lactic acid, and acetic acid, and by competitive exclusion [6]. 

Prebiotics are considered preventative agents since they select for gastrointestinal microbiota, which not only benefit the host but also can serve as a barrier to pathogen colonization [7]. Growing evidence obtained from in vitro animal and human studies strongly suggests the immunomodulatory effect of arabinogalactan (ARB), which belongs to a major group of carbohydrates known as hemicelluloses [8]. ARB has a wide range of biological properties and activities, such as the protection of gastrointestinal mucosa [9], enhancement of gut health by stimulating the microbiome establishment [10], alleviating the stress induced by gastrointestinal dysfunctions [10], and also improving vascular permeability and enhancing the immune function (Dion et al., 2016). There are no published data about the influence of the LAB LUHS245 strain and ARB combination on newborn calves’ health. Therefore, the aim of this study was to evaluate the influence of the *Lactobacillus uvarum* LUHS245 strain, ARB, and their combination on health parameters, including the blood biochemistry and fecal microbiology of newborn calves. Our hypothesis is that a combination of different compounds of different origins and mechanisms of action (LAB as an antimicrobial agent and ARB as a prebiotic for good microbiota stabilization), can lead to an improvement in the health parameters of newborn calves. An additional aim was to evaluate the antimicrobial properties of LUHS245, ARB, and their combination against a variety of pathogenic and opportunistic bacterial strains (*Klebsiella pneumoniae, Pseudomonas aeruginosa* 17-331, *Acinetobacter baumanni* 17-380, *Proteus mirabilis*, MRSA M87fox, *Enterococcus faecalis* 86, *Enterococcus faecium* 103, *Bacillus cereus* 18 01, and *Streptococcus mutans*). We also determined the resistance to antibiotics of LUHS245 strain in this research. 

## 2. Materials and Methods 

### 2.1. Lactobacillus uvarum LUHS245 Purification, Isolation, Identification, and Characterization

*Lactobacillus uvarum* LUHS245 strain was isolated from spontaneously-fermented wholemeal rye (328 s, falling number ˃ 67.8% starch, ash 1.30%) obtained from the Litagra group company (Kedainiai, Lithuania). Spontaneous fermentation was performed using the following protocol: rye flour (100 g) was mixed with 1% acetic acid (Sigma-Aldrich, Taufkirchen, Germany), 1% NaCl (Sigma-Aldrich, Taufkirchen, Germany) and 150 mL tap water, and fermented for 48 h in oven at 30 °C, followed by the addition of 50 g rye flour (Litagra group, Kedainiai, Lithuania) and water (50 mL) and fermented further for another 24 h at 30 °C, and used for the isolation of LAB afterwards. 

Purification of LAB cells was performed according to the method described by Kiss et al. [11]. Molecular fingerprinting of the final strains was done by rep-typing with the primer GTG5 (5’-GTG GTG GTG-3’) [12]. Polymerase chain reaction (PCR) was carried out in a Mastercycler (Eppendorf, Hamburg, Germany) according to Song et al. [13]. The resulting (GTG) 5-PCR fingerprints were analyzed using the BioNumerics v4.0 software package (Applied Maths, Sint-Martens-Latem, Belgium). 

Carbohydrate metabolism of the strains was determined by using API 50 CH Kits (BioMerieux, Marcy-l’Etoile, France) according to the manufacturer’s instructions. Gas production was detected by Durham tube method in MRS broth (Oxoid CM361, Basingstoke, Hampshire, England) for 24 h at 30 °C. The growth performance of strains was monitored at 10, 30, 37, and 45 °C for 24 h in a MRS broth using a Thermo Bioscreen C automatic turbidometer (Labsystems, Helsinki, Finland). The ability of the strains to survive at low pH was evaluated in acidified with HCl (Sigma-Aldrich, Taufkirchen, Germany) up to pH 2.5 MRS broth with tween 80 (Biolife, Milano, Italia) [14]. All analyses were carried out in triplicate.

### 2.2. Evaluation of Lactobacillus uvarum LUHS245 Strain, Arabinogalactan, and their Combination Antimicrobial Activities 

Arabinogalactan (D-galactose and L-arabinose in a 7.5:1 ratio) used in this study was extracted from Larix spp. wood (Siberia, Russia), and purchased from SME Rokiskio pragiedruliai (Rokiskis, Lithuania).

Antimicrobial activities of the *Lactobacillus uvarum* LUHS245 strain, ARB, and their interaction were determined against *Klebsiella pneumonia, Salmonella enterica* 24 SPn06, *Pseudomonas aeruginosa* 17-331, *Acinetobacter baumanni* 17-380, *Proteus mirabilis*, Methicillin-resistant *Staphylococcus aureus* M87fox (MRSA), *Enterococcus faecalis* 86, *Enterococcus faecium* 103, *Bacillus cereus* 18 01, and *Streptococcus mutans*. The used pathogenic and opportunistic bacterial strains were attained from the Lithuanian University of Health Sciences’ (Kaunas, Lithuania) collection.

Antimicrobial activity of the LUHS245 strain, ARB and their mix was tested by an agar-well diffusion assay by measuring the diameter of inhibition zones (DIZ, mm) [15]. For this purpose, 0.5 McFarland Unit density suspension (~10^8^ CFU mL^−1^) of each pathogenic strain was inoculated onto the surface of cooled Mueller–Hinton agar (Oxoid, Basingstoke, UK) using sterile cotton swabs. Wells of 6 mm in diameter were punched in the agar and filled with 50 µL of LAB cultivated in MRS broth (Oxoid, UK). Before the experiment, ARB was diluted with sterile physiological solution and multiplied in MRS broth LUHS245 (1 g of the ARB with 2 mL of the physiological solution and 2 mL of LUHS245). The experiments were repeated three times and the average of DIZ was calculated.

### 2.3. Evaluation of the Lactobacillus uvarum LUHS245 Strain Resistance to Antibiotics 

The minimum inhibitory concentrations (MICs) of gentamycin (GEN), tetracycline (TET), erythromycin (ERY), amoxicillin (AML), and trimethoprim (TM) were determined by the micro-dilution method [16]. The MICs were evaluated as the lowest concentrations of given antibiotics at which no growth of the test organisms was observed. Microbiological cut-off values were used as the interpretative criteria for susceptibility testing according to the EFSA Panel on Additives and Products or Substances used in Animal Feed Breakpoint (FEEDAP) guidelines [16].

### 2.4. In Vivo Experiment with Newborn Calves 

A total number of 48 Holstein female calves were randomly allocated in four homogeneous groups (each group consisted of 12 calves) on the basis of body weight on day of birth, and the experiment started from the second day of life. Calves received first colostrum by their dams during the first day after birth and were enrolled in the study on day 2 of life. Calves of the control group (CON) were fed with a standard milk replacer diet and colostrum only. Calves of the treated groups were fed with the same diet supplemented either with 50 mL of the LUHS245 strain (≥7.5 log_10_ CFU mL^−1^) (LHU group), 30 g of ARB (ARA group), or with 50 mL of LUHS245 plus 30 g ARB (BOTH group).

All supplements (separate and in combination) were added and mixed in the milk replacer (22.5% crude protein, 18% fat, 9.0% ashes, 1.75% lysine, 0.55% methionine, and 0.50% cysteine on a dry matter basis and the milk powder (130 g L^−1^ reconstituted in hot water (65 °C) and fed at a temperature of 39 °C in a bucket) during the morning feeding. Each calf was placed in an individual outdoor box (2.00 × 1.25 m), with free access to warm water. Calves were fed individually once a day (7:00 a.m.) with non-medicated milk replacer (8–10 L per calf per day). 

Calves were bled (5 mL) aseptically from jugular vein into vacuum blood tubes (BD Vacutainer®, Weymouth, UK) at days 2 and 14 of the experiment before the morning feeding. Samples taken on day 2 were before the treatment started and were used as baseline measurements. Tubes containing lithium heparin were used to study blood gas, and the tubes with clot activator were used for biochemical examination of blood. The blood parameters of calves, including albumin (Alb.), urea, base excess of extra cellular fluid (BE_ecf_),O_2_ saturation, pH, carbon dioxide partial pressure (_P_CO_2_), arterial oxygen partial pressure (_P_O_2_), Na, K, ionized calcium (iCa), glucose (Glu), lactates, hematocrit (Hct), base excess of blood (BE(b)), bicarbonate (HCO_3_), total amount of CO_2_ (_T_CO_2_), and hemoglobin (Hb) were analysed using an automatic blood gas analyzer (EPOC, Ottawa, Canada). After collection of blood into the vacutainer tubes with clot activator, samples were centrifuged (Hettich Universal, Tuttlingen, Germany) at 6000× *g* for 10 min to obtain plasma and serum. To evaluate health status, biochemical variables of the blood, as well as counts of LAB, total number of cultivable bacteria, *Enterobacteriaceae*, and the number of yeasts and molds were evaluated before and after the experiment (on days 2 and 14 of the calves’ life). Concentrations of serum alanine aminotransferase (AST) in calves’ blood were measured using an automated analyzer Hitachi 705 (Hitachi, Tokyo, Japan) and DIAS (Diagnostic Systems GmbH, Germany) reagents.

The calves’ fecal samples were collected on days 2 and 14 of life directly from the anus into clean plastic vials immediately after a gloved, lubricated finger was gently passed through the anus to massage the rectal wall and to stimulate rectal evacuation, stored in vials (+4 °C) with a transport medium (Faecal TM enteric Plus, Oxoid, Basingstoke, UK) and analyzed on the same day. De Man, Rogosa, and Sharpe (MRS) agar (Oxoid Ltd., Basingstoke, UK) was used for the determination of LAB. The violet red bile glucose (VRBG) agar (Oxoid Ltd., Basingstoke, UK) was used for the determination of the total count of enterobacteria. The plate count agar (Biolife Italiana Srl, Milan, Italy) was used for the determination of the total aerobic bacteria and facultative anaerobes, and the dichloran rose bengal chloramphenicol (DRBC) agar (Liofilchem, Milan, Italy) was used for the yeast and mold (Y/M) count determination. The results were expressed as a log_10_ of CFU g^−1^ of a sample. 

### 2.5. In Vivo Experiment Ethical Guidelines

The calves were hosted indoors, being individually tethered and cared for in accordance with the Lithuanian State Food and Veterinary Service Requirements. Research was carried out in accordance with the 6 November 1997 Republic of Lithuania Act covering animal care and maintenance, and the appropriate legal act, 8-500 (Valstybės Žinios, (Official Gazette) No 130-6595: 2012) [17].

### 2.6. Statistical Analysis

All in vitro experiments of LAB characteristics were performed in at least two independent experiments. In the analysis of variance (ANOVA), treatment was considered as fixed effect, and plate within the experimental day as random effect. The means and standard deviations of the data were computed. All analytical determinations of calves’ blood and fecal parameters were performed in triplicate. Data were subjected to two-way ANOVA using statistical package SPSS for Windows (Ver. 15.0, SPSS, Chicago, Illinois, USA). Baseline measurements were used as covariates to take the experimental conditions into account. The mean values were compared using Duncan’s multiple range test with significance level defined at *p* ≤ 0.05. 

## 3. Results and Discussion

### 3.1. Characteristics of the Lactobacillus uvarum LUHS245 Strain 

**Figure 1:**
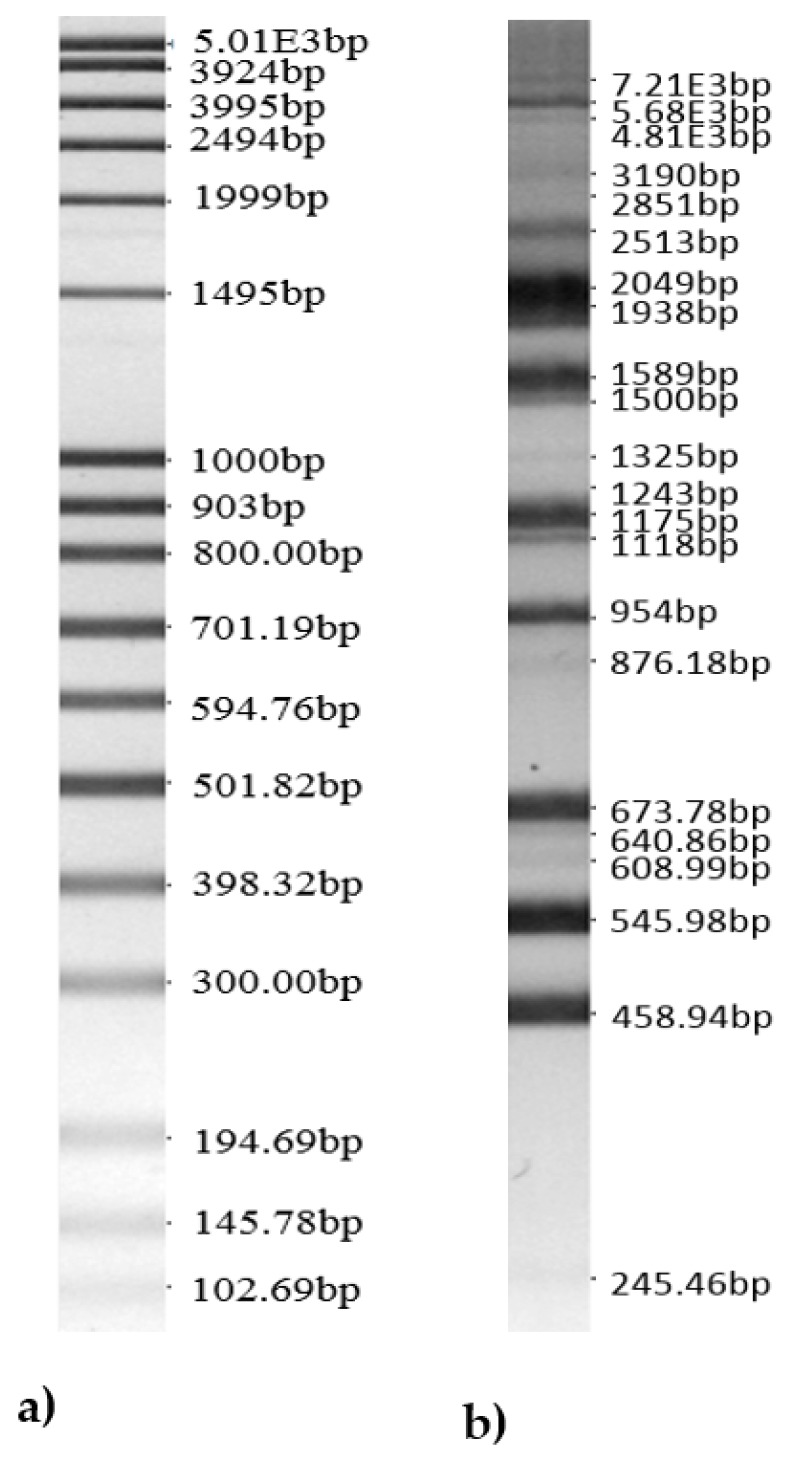
Bands of the isolated *Lactobacillus uvarum* LUHS245 genus. (**a**) 100bp DNA-ladder extended and (**b**) bands of isolated *Lactobacillus uvarum* LUHS245 genus).

Data of the analysis of the newly-isolated *Lactobacillus uvarum* bands by using the *BioNumerics* v4.0 software package are shown in Figure 1. 

*Lactobacillus uvarum* carbohydrate metabolism, gas production, tolerance to temperature and low pH conditions (pH 2.5 for 2 h of incubation) are shown in Table 1. LUHS245 showed a high activity of the carbohydrate substrate tested, such as D-glucose, D-fructose, D-mannose, D-mannitol, methyl-αD-glucopyranoside, N-acetylglucosamine, amygdalin, arbutin, esculin, salicin, D-cellobiose, D-maltose, D-sucrose, D-trehalose, gentiobiose, and D-turanose fermentation (Table 1).

Carbohydrate fermentation profiles of *Lactobacillus* spp. utilization of many oligosaccharides from different categories appears to be a ubiquitous feature of lactobacilli [18]. This energy source is one of the most important aspects for newborn calves, so commercial milk replacers, including this milk replacer, contain on a dry matter basis, high levels of lactose (36%–40%), fat (30%–40%), and milk protein (28%–32%) [19], whereas in contrast, LUHS245 does not utilize lactose and fat, therefore, the availability of key energy-releasing nutrients (i.e., lactose and fat) is not reduced in calves fed milk replacers.

After 2 h of incubation at pH 2.5, the count of viable LUHS245 cells was 7.55 ± 0.1 log_10_ CFU mL^−1^. The capacity of *Lactobacillus* strains to act as probiotics is also determined by their ability to survive in the low pH of the stomach and in the high concentration of bile salt of the gastrointestinal tract [20]. Gastrointestinal conditions along the digestive tract are the main stress to which probiotics administrated orally are exposed because they must survive these adverse conditions and arrive alive to the intestine. Adhesion to the epithelium has been considered one of the key criteria for the characterization of probiotics because it extends their residence time in the intestine and can influence the health of the host by modifying the local microbiota or modulating the immune response [21]. According to Bengoa et al. [21] after gastrointestinal passage all the *Lactobacillus paracasei* strains isolated from kefir, have increased their ability to adhere to mucin and epithelial cells in vitro to exert their probiotic action. The digestive enzymes allow highly efficient digestion of milk proteins, lactose, and triacylglycerides, and smaller digestion of non-milk proteins or polysaccharides such as starch [22].

The tested strain showed the highest growth at 30 °C and 37 °C, and was not detected at 45 °C. The normal temperature of the gastrointestinal tract is in the range of 39–39.5 °C [23], but did not increase to the temperatures where LUHS245 cannot survive and multiply in the gut. The tested strains showed no gas production (Table 1). 

### 3.2. Antimicrobial Properties of Arabinogalactan, the Lactobacillus uvarum LUHS245 Strain, and their Combination 

Antimicrobial activity of the LUHS245 strain, ARB and their interaction is presented in Table 2. The LUHS245 inhibited the growth of all the tested pathogenic strains. The DIZ toward pathogenic strains varied between 13.0 mm and 21.3 mm for LUHS245, and the highest antimicrobial activity was observed against *B. cereus* (inhibition zone diameter was 21.3 ± 0.5 mm). Antimicrobial activity is a very important criterion for selection of starter and probiotic culture as natural antagonists of potentially harmful bacteria [24]. According to our previous studies the LUHS245 strain in liquid medium inhibited the growth of *Klebsiella pneumonia, Salmonella enterica, Pseudomonas aeruginosa, Acinetobacter baumannii, Proteus mirabili*, methicillin-resistant *Staphylococcus aureus, Enterococcus faecalis, Enterococcus faecium, Bacillus cereus, Streptoccocus mutans, Enterobacter cloacae, Citrobacter freundii, Staphylococcus epidermis, Staphylococcus haemolyticus,* and *Pasteurella multocida* [25]. LAB produce bacteriocins and antimicrobial peptides that have killing activity principally against other relatively closely-related bacteria. Effects of bacteriocins include controlling the growth of an increasingly-heterogeneous variety of pathogens, including Gram-negative multidrug resistant bacteria [26]. Moreover, LAB produce lactic and acetic acids and in smaller amounts, formic acid, free fatty acids, ammonia, ethanol, hydrogen peroxide, diacetyl, acetoin, 2,3-butanediol, acetaldehyde, benzoate, and bacteriolytic enzymes and have an antagonistic effect towards Gram-negative pathogens that could be related to the production of these compounds [25]. ARB inhibited the growth of *Proteus mirabilis*, *MRSA* M87fox, and *Bacillus cereus* 18 01 (17.0 ± 0.3, 11.0± 0.1, and 10.0 ± 0.2, respectively). LUHS245 and ARB combination inhibited the growth of four pathogenic bacterial strains (DIZ against *Salmonella enterica* 24 SPn06 was 9.0 ± 0.1 mm, against *Acinetobacter baumanni* 17-380 it was 14.0 ± 0.2 mm, against *MRSA* M87fox it was 9.0 ± 0.2 mm, and against *Enterococcus faecium* 103 it was 14.0 ± 0.3 mm). According to the results obtained, in most of the cases, higher antimicrobial activity was shown by LUHS245 alone or in the combination with ARB, compared to ARB alone. Disparity in the antagonism activity against different pathogens indicates that probiotic strains are highly pathogen specific and prerequisite for probiotic potential. Also, larch ARB was approved by the Food and Drug Administration in 1965 for direct addition to food/feed and gained generally recognized as safe (GRAS) notification in 2000 [27,28]. Soluble carbohydrates such as ARB recognized by the bacterial lectins block the adhesion of the bacteria to animal cells in vitro. Moreover, they have also been shown to protect against experimental infection by lectin-carrying bacteria in different organs of mammals such as mice, rabbits, calves, and monkeys [29]. Preventing bacterial adhesion to host cells is a provocative and alternative approach to traditional antibiotic treatments given the increasing microbial resistance [30]. Studies about the antimicrobial activity of *Lactobacillus uvarum* are scarce, and our study showed that LUHS245 strain is very promising as an antimicrobial agent for feed preparation. However, more research is needed to explain the mechanism of the synergism between the plant and microbial inhibiting compounds.

### 3.3. Lactobacillus uvarum LUHS245 Resistance to Antibiotics

The LUHS245 strain was considered non-resistant to all the tested antibiotics (gentamicin tetracycline, erythromycin, amoxicillin, and trimethoprim), when the MIC (mg mL^−1^) values obtained were the same or lower than the recommended breakpoint value, defined at species level by the FEEDAP [16] (Table 2). According to Georgieva et al. [24], most of *Lactobacilli* strains were susceptible toward ampicillin, gentamicin, erythromycin, trimethoprim, and tetracycline. Antibiotics are widely used in food-producing animals. It is believed that using these antibiotics contributes to the emergence of antibiotic-resistant bacteria present in the intestinal microflora [31]. Then, these antibiotic-resistant bacteria can transfer resistance factors to other pathogenic bacteria through the exchange of genetic material. One of the safety considerations in probiotics is verifying that a potential probiotic strain does not contain transferable resistance genes [32].

### 3.4. The Influence of Arabinogalactan, the Lactobacillus uvarum LUHS245 Strain, and their Combination on Newborn Calves’ Health Parameters

The influence of the supplementation with LUHS245 strain and ARB, alone or together, on the feces microbiological parameters of calves are presented in Table 3. Results of the ANOVA indicated that there is an interactive effect (*p* ≤ 0.0001) of feeding duration and feed additives on the microbiological parameters of calves’ feces samples. For example, it was found that groups LUH, ARA, and BOTH had significantly lower enterobacteria counts (by 13.3%, 27.1%, and 28.0%, respectively,) in feces on day 14, compared with the CON group. Also, calf groups LUH, ARA, and BOTH, after day 14, had a significantly higher LAB count (by 45.8%, 9.9%, and 41.2%, respectively). According to Kawakami et al. [33], feeding with LAB significantly increased the number of fecal LAB of calves suggesting that the bacteria have a probiotic ability and improve the balance of enteric microbial flora. These beneficial bacteria exclude gut pathogens through competitive exclusion by suppressing growth, thus reducing toxic fermentation products. This is accomplished by preventing the adhesions of pathogens to mucosa by competing with the sugar receptors [34]. Also, the efficiency of ARB in veterinary medicine has been proven [35]. Consumption of prebiotics such as ARB has been shown to increase beneficial bacteria such as *Bifidobacteria* and *Lactobacilli* in the intestine and decrease bouts of diarrhea. The production of these bacteria are beneficial due to their ability to reduce gut pH, stimulate immunoglobulin production, and produce short chain fatty acids (SCFA) such as butyrate and propionate [36]. These SCFA are critical to the health of the colon. They protect the intestinal lining against disease and cancer-promoting agents by providing the main energy source for the colonic cells and increasing beneficial LAB [37]. Prebiotics themselves have a positive influence on immune parameters in the gut-associated lymphoid tissues, secondary lymphoid tissues, and peripheral circulation [38]. Prebiotics may promote T Helper 1- and regulatory T cell-dependent immune responses over T helper 2 responses [39]. In cell and animal models, larch ARB is capable of enhancing natural killer cells and macrophages, as well as the secretion of pro-inflammatory cytokines [8]. 

### 3.5. Influence of the Lactobacillus uvarum LUHS245 Strain, Arabinogalactan, and their Combination on Newborn Calves’ Blood Parameters

Dietary supplementation of LUHS245 and ARB improved some hematological profile values of newborn calves’ blood parameters (Table 4). The significant lower serum alanine aminotransferase (AST) activity in LUH- and ARA-fed groups (by 26.05% and 64.06%, respectively, lower after day 14) was established, compared with samples of the same groups at the beginning of experiment. The activity of hepatic AST is increased during pathological processes and these enzymes are released into the bloodstream [40]. An AST value between 5 and 40 U L^−1^ is considered normal [41] and the results of our study fell within the normal range after 14 days feeding for both LUHS245 and ARB groups but was higher in the control group. Lactate plays a significant role in neonatal acidosis subsequent to asphyxia. It is responsible for metabolic acidosis and persists in blood in increased concentrations considerably longer than CO_2_ [42]. The concentration of lactate decreased in all groups, however, we found a significant higher reduction (by 67.6%) in with ARA-fed group. Also, LUH and BOTH treatments significantly reduced (by 9.9% and 45.4%, respectively,) lactate concentration in calves’ blood after day 14. Lorenz [43] found significantly higher blood D-lactate concentrations in diarrheic calves with ruminal acidosis than in those without this disorder. These authors suggested that ruminal acidosis might be a complication of the D-lactic acidosis caused by diarrhea and subsequent malabsorption rather than vice versa. Our group previously showed that fermented potato juice, with LAB, administered to calves for 14 days, reduced the risk of acidosis, and reduced lactate concentrations as well as liver lesions and *E. coli* in the feces [44]. Probiotics seems to be effective in preventing or treating ruminal acidosis in calves. Application of *Propionibacterium* P63, *L. plantarum* strain 115, and *L. rhamnosus* strain 32 to the rumen directly via a rumen cannula at the rate of 1 ×10^11^ CFU per animal per day was effective in stabilizing rumen pH and preventing acidosis in sheep [45]. We also observed significantly lower _P_O_2_ concentration in LUH, ARA, and BOTH groups (by 42.9%, 57.9%, and 39.3%, respectively,) compared with measurements before the experiment. However, it should be mentioned that this concentration was higher than the physiological normal concentration (60 mmHg). Results of the ANOVA indicated that there is a significant effect (*p* < 0.0001) due to the calves’ treatment duration, as well as interaction of treatment duration and additive used on most of the tested blood parameters (except for pH, Na, _T_CO_2_, and Hct fraction). 

## 4. Conclusions

LUHS245 strain effectively inhibited the growth of Gram-positive and Gram-negative pathogenic bacteria, *Salmonella enterica* 24 SPn06 and *Bacillus cereus* 08 01, as well as being non-resistant to all the tested antibiotics. LUHS245, ARB, and their combination had some effect on newborn calves’ fecal and blood parameters such as increased LAB count (on average by 33.20%) and reduced enterobacteria count (on average by 22.88%) in feces, as well as reducing lactate concentration (on average by 44.69%) in blood. More research with larger cohorts of calves is needed to validate these findings and to also explain the mechanism of action of the tested compounds.

## Figures and Tables

**Table 1:** *Lactobacillus uvarum* LUHS245 carbohydrate metabolism, gas production, and tolerance to temperature and low pH conditions.

Carbohydrate		Interpretation of LAB Growth in API 50 CH System
Glycerol		-
Erythritol		-
D-arabinose		-
L-arabinose		-
D-ribose		-
D-xylose		-
L-xylose		-
D-adonitol		-
Methyl-ßd-xYlopiranoside		-
D-galactose		-
D-glucose		+++
D-fructose		+++
D-mannose		+++
L-sorbose		+
L-rhamnose		-
Dulcitol		-
Inositol		-
D-mannitol		+++
D-sorbitol		-
Methyl-αD-mannopyranoside		-
Methyl-αD-glucopyranoside		+++
N-acetylglucosamine		+++
Amigdalin		+++
Arbutin		+++
Esculin		+++
Salicin		+++
D-cellobiose		+++
D-maltose		+++
D-lactose		-
D-melibiose		-
D-sucrose		+++
D-trehalose		+++
Inulin		-
D-melezitose		-
D-raffinose		-
Starch		-
Glycogen		-
Xylitol		-
Gentiobiose		+++
D-turanose		+++
D-lyxose		-
D-tagatose		-
D-fucose		-
L-fucose		-
D-arabitol		-
L-arabitol		-
Potassium gluconate		-
Potassium 2-ketogluconate		-
Potassium 5-ketogluconate		-
Gas production (+/-)		-
Tolerance to temperature	10 °C 30 °C 37 °C 45 °C	-
		++
		++
		-
pH 2.5	After 0 h, LAB count, log_10_ CFU mL^−1^ After 2 h, LAB count, log_10_ CFU mL^−1^	9.03 ± 0.2
		7.55 ± 0.1

Interpretation of LAB growth in API 50 CH system. LAB: lactic acid bacteria, +++ = high growth (yellow), ++ = medium growth (green), + = little growth (dark green), and − = no growth (blue).

**Table 2:** Inhibition of the growth of pathogenic bacteria by *Lactobacillus uvarum, L. uvarum*–arabinogalactan mix, and arabinogalactan powder and their resistance to antibiotics.

Microorganisms	Zone of Inhibition (mm)			Antibiotics	Resistance to Antibiotics	
	*L. uvarum*	*L. uvarum* and Arabinogalactan Mix	Arabino-galactan		*L. uvarum*	FEEDAP Breakpoint (mg mL ^−1^)
					MIC (mg mL^−1^)	
*Klebsiella pneumoniae*	14.0 ± 0.2	-	-			
*Salmonella enterica*	13.0 ± 0.3	9.0 ± 0.1	-	GEN	16.0 ± 0.2	16
*Pseudomonas aeruginosa* 17-331	16.2 ± 0.3	-	-			
*Acinetobacter baumanni* 17-380	15.0 ± 0.4	14.0 ± 0.2	-	TET	0.75 ± 0.4	8
*Proteus mirabilis*	15.0 ± 0.1	-	17.0 ± 0.3			
*MRSA* M87fox	16.0 ± 0.2	9.0 ± 0.2	11.0 ± 0.1	ERY	0.0016 ± 0.3	1
*Enterococcus faecalis* 86	16.0 ± 0.3	-	-			
*Enterococcus faecium* 103	20.0 ± 0.2	14.0 ± 0.3		AML	0.016 ± 0.1	n.r.
*Bacillus cereus* 18 01	21.3 ± 0.5	-	10.0 ±0.2			
*Streptococcus mutans*	15.0 ± 0.1	-	-	TM	0.75 ± 0.2	n.r.

Values are mean of three replicate analyses. GEN: gentamicin, TET: tetracycline, ERY: erythromycin, AML: amoxicillin, TM: trimethoprim, MIC: minimum inhibitory concentration, and n.r.: not required. FEEDAP: Panel on Additives and Products or Substances used in Animal Feed Breakpoint (EFSA Journal 2012).

**Table 3:** Fecal microbiological parameters of calves fed milk replacer only (CON), or supplemented with *L. uvarum* LUHS245 strain (LUH) or arabinogalactan (ARA) alone, and together (BOTH).

Variable	Day	Treatments				*p*-Value
		CON	LUH	ARA	BOTH	Day × Treat. Int.
TCM	Baseline	6.15 ± 0.02 ^Ab^	7.73 ± 0.02 ^Ad^	6.92 ± 0.01 ^Ac^	5.26 ± 0.01 ^Aa^	0.0001
	14	8.21 ± 0.01 ^Bb^	8.34 ± 0.03 ^Bc^	8.46 ± 0.03 ^Bd^	7.80 ± 0.05 ^Ba^	
LAB	Baseline	3.66 ± 0.01 ^Aa^	4.08 ± 0.03 ^Ab^	5.84 ± 0.02 ^Ad^	4.13 ± 0.01 ^Abc^	0.0001
	14	6.15 ± 0.04 ^Ba^	7.53 ± 0.04 ^Bd^	6.48 ± 0.02 ^Bb^	**7.02 ± 0.03 ^Bc^**	
TCE	Baseline	4.81 ± 0.01 ^Aa^	6.55 ± 0.01 ^Ac^	6.78 ± 0.03 ^Bd^	6.46 ± 0.03 ^Bb^	0.0001
	14	6.92 ± 0.02 ^Bd^	5.68 ± 0.06 ^Bc^	4.94 ± 0.03 ^Ab^	**4.65 ± 0.04 ^Aa^**	
Y/F	Baseline	3.45 ± 0.02 ^Aa^	6.97 ± 0.03 ^Ad^	5.89 ± 0.01 ^Bc^	3.94 ± 0.01 ^Ab^	0.0001
	14	5.54 ± 0.03 ^Ba^	5.87 ± 0.03 ^Ab^	5.65 ± 0.02 ^Aa^	6.19 ± 0.03 ^Bc^	

TCM: total count of aerobic and facultative anaerobic microorganisms, LAB: lactic acid bacteria count, TCE: total count of enterobacteria, Y/F: yeasts/fungi; Treat. Int.— treatment interaction; ^A,B^ different capitals indicate significant time-related differences (*p* < 0.05). ^a–d^: different letters indicate differences among treatments (*p* < 0.05). Data are presented as mean ± SE (n = 12/group); Baseline measurements were done on day 2, before the start of the feeding experiment; The most effective activities of BOTH appear in bold.

**Table 4:** Blood parameters of calves fed with milk replacer only (CON) or supplemented with LUHS245 strain (LUH) or arabinogalactan (ARA) alone and together (BOTH).

Variable	Day	Treatments				*p*-Value
		CON	LUH	ARA	BOTH	Day × Treat. Int.
Alb.	Baseline	26.55 ± 0.50 ^Aa^	27.13 ± 0.11 ^Aa^	26.40 ± 0.82 ^Aa^	34.85 ± 0.73 ^Aa^	0.0001
	14	26.84 ± 0.93 ^Aa^	27.83 ± 0.72 ^Aa^	36.81 ± 0.74 ^Bc^	38.92 ± 0.95 ^Bd^	
Urea	Baseline	3.35 ± 0.02 ^Ba^	2.82 ± 0.07 ^Aa^	3.63 ± 0.01 ^Ba^	3.34 ± 0.09 ^Bb^	0.0001
	14	2.26 ± 0.03 ^Ab^	3.11 ± 0.02 ^Bc^	2.21 ± 0.01 ^Ad^	2.52 ± 0.03 ^Aa^	
AST	Baseline	54.67 ± 1.01 ^Ab^	59.50 ± 1.02 ^Bc^	79.33 ± 1.47 ^Ba^	37.34 ± 1.97 ^Ab^	0.0001
	14	50.67 ± 1.44 ^Ad^	44.00 ± 1.55 ^Ac^	28.51 ± 1.72 ^Aa^	39.00 ± 1.93 ^Aa^	
pH	Baseline	7.45 ± 0.02 ^Aa^	7.41 ± 0.05 ^Aa^	7.42 ± 0.07 ^Aa^	7.43 ± 0.08 ^Aa^	0.956
	14	7.40 ± 0.08 ^Aa^	7.39 ± 0.04 ^Aa^	7.37 ± 0.02 ^Aa^	7.39 ± 0.01 ^Aa^	
_P_CO_2_ mmHg	Baseline	41.00 ± 0.91 ^Ab^	50.15 ± 1.24 ^Ac^	43.20 ± 1.58 ^Ab^	37.75 ± 1.65 ^Aa^	0.056
	14	52.28 ± 0.53 ^Bb^	48.25 ± 0.61 ^Aa^	57.85 ± 0.22 ^Bc^	51.48 ± 0.41 ^Bb^	
_P_O_2_ mmHg	Baseline	165.23 ± 2.72 ^Bc^	133.95 ± 3.42 ^Ba^	158.97 ± 3.61 ^Ba^	135.97 ± 4.64 ^Bc^	0.0001
	14	103.90 ± 2.91 ^Ad^	76.53 ± 3.22 ^Ab^	66.9 ± 2.23 ^Aa^	82.53 ± 2.34 ^Aa^	
HCO_3_ mmol/L	Baseline	27.85 ± 0.93 ^Aa^	31.20 ± 0.88 ^Ab^	28.00 ± 0.51 ^Aa^	30.13 ± 0.78 ^Ab^	0.0001
	14	32.40 ± 0.94 ^Bab^	29.13 ± 0.83 ^Aa^	33.03 ± 0.82 ^Bc^	31.18 ± 0.91 ^Ba^	
BE (ecf)	Baseline	3.77 ± 0.05 ^Ab^	6.48 ± 0.02 ^Ba^	3.58 ± 0.03 ^Ac^	3.11 ± 0.02 ^Ab^	0.0001
	14	7.55 ± 0.01 ^Bb^	4.15 ± 0.03 ^Ab^	7.68 ± 0.02 ^Ba^	6.25 ± 0.05 ^Ba^	
O_2_ saturation	Baseline	92.65 ± 1.62 ^A;a^	86.72 ± 1.51 ^Aa^	98.42 ± 2.35 ^Ba^	94.56 ± 2.64 ^Ba^	0.0001
	14	93.60 ± 2.48 ^A;b^	91.52 ± 3.63 ^Bb^	88.12 ± 1.93 ^Aa^	87.88 ± 2.92 ^Aa^	
Na, mmol/L	Baseline	139.67 ± 4.51 ^A;a^	137.50 ± 3.92 ^Aa^	139.00 ± 3.23 ^Aa^	136.54 ± 2.91 ^Aa^	0.438
	14	137.83 ± 3.73 ^A;a^	138.50 ± 3.43 ^Aa^	137.00 ± 2.54 ^Aa^	141.00 ± 5.39 ^Aa^	
K, mmol/L	Baseline	5.07 ± 0.03 ^A;c^	4.57 ± 0.03 ^Aa^	4.80 ± 0.02 ^Ab^	11.17 ± 0.04 ^Bd^	0.0001
	14	5.52 ± 0.09 ^Bb^	4.90 ± 0.01 ^Bb^	5.07 ± 0.07 ^Bb^	4.75 ± 0.01 ^Ab^	
iCa, mmol/L	Baseline	1.24 ± 0.01 ^Aa^	1.27 ± 0.02 ^Ab^	1.28 ± 0.03 ^Ab^	1.81 ± 0.02 ^Bd^	0.0001
	14	1.25 ± 0.02 ^Aa^	1.26 ± 0.01 ^Aa^	1.26 ± 0.03 ^Aa^	1.26 ± 0.02 ^Aa^	
_T_CO2, mmol/L	Baseline	29.10 ± 1.21 ^ab^	32.72 ± 2.07 ^b^	29.33 ± 2.61 ^ab^	23.98 ± 3.65 ^a^	0.154
	14	34.02 ± 2.03 ^a^	30.60 ± 3.61 ^a^	34.80 ± 2.45 ^a^	32.75 ± 2.36 ^a^	
Hct fraction	Baseline	26.33 ± 3.15 ^Aa^	27.00 ± 4.03 ^Aab^	26.83 ± 2.01 ^Aa^	26.09 ± 1.01 ^Aa^	0.604
	14	25.50 ± 2.30 ^Aa^	23.83 ± 1.23 ^Aa^	25.83 ± 1.53 ^Aa^	26.50 ± 1.25 ^Aa^	
Hb, g/L	Baseline	8.92 ± 0.01 ^Ba^	9.23 ± 0.02 ^Bc^	9.18 ± 0.04 ^Bb^	9.11 ± 0.03 ^Ab^	0.0001
	14	8.67 ± 0.03 ^Ab^	8.08 ± 0.05 ^Aa^	8.73 ± 0.04 ^Ab^	9.08 ± 0.02 ^Ac^	
BE(b)	Baseline	3.40 ± 0.02 ^Ab^	5.72 ± 0.03 ^Bd^	3.17 ± 0.05 ^Aa^	5.11 ± 0.04 ^Ac^	0.0001
	14	6.58 ± 0.03 ^Bc^	3.62 ± 0.04 ^Aa^	6.63 ± 0.05 ^Bc^	5.40 ± 0.04 ^Bb^	
Glu, mmol/L	Baseline	6.37 ± 0.01 ^Ab^	7.17 ± 0.03 ^Bb^	7.42 ± 0.04 ^Bd^	6.16 ± 0.05 ^Ba^	0.031
	14	6.05 ± 0.01 ^Abc^	5.92 ± 0.03 ^Ab^	5.93 ± 0.04 ^Ad^	5.21 ± 0.06 ^Ab^	
Lactate, mmol/L	Baseline	6.19 ± 0.04 ^Bc^	4.25 ± 0.03 ^Ba^	6.24 ± 0.02 ^Bb^	5.88 ± 0.04 ^B^	0.0001
	14	3.24 ± 0.06 ^Ab^	3.83 ± 0.03 ^Ac^	2.02 ± 0.04 ^Aa^	3.21 ± 0.01 ^Ab^	

Alb: albumin, AST: aminotransferase, BE_ecf_: base excess extra cellular fluid, _P_CO_2_: carbon dioxide partial pressure, _P_O_2_: arterial oxygen partial pressure, iCa: ionized calcium, Glu: glucose, Hct hematocrit, BE(b): base excess (blood), HCO_3_: bicarbonate, _T_CO_2_: total amount of CO_2_, and Hb: hemoglobin; Treat. Int.— treatment interaction; ^A,B^ different capitals indicate significant time-related differences (*p* < 0.05). ^a-d^ different letters indicate differences among treatments (*p* < 0.05). Data are presented as mean ± SE (n = 12/group); Baseline measurements were done on day 2, before the start of the feeding experiment; The most effective activities of BOTH appear in bold.
